# Improved estimation of motion blur parameters for restoration from a single image

**DOI:** 10.1371/journal.pone.0238259

**Published:** 2020-09-01

**Authors:** Wei Zhou, Xingxing Hao, Kaidi Wang, Zhenyang Zhang, Yongxiang Yu, Haonan Su, Kang Li, Xin Cao, Arjan Kuijper

**Affiliations:** 1 School of Information Science and Technology, Northwest University, Xi’an, P.R.China; 2 Xi’an Institute of Optics and Precision Mechanics of CAS, Xi’an, P.R.China; 3 Department of Electrical and Automatic Engineering, East China Jiaotong University, Nanchang, P.R.China; 4 School of Electronic Engineering, Xidian University, Xi’an, P.R.China; 5 Fraunhofer IGD, Darmstadt, Germany; Politechnika Slaska, POLAND

## Abstract

This paper presents an improved method to estimate the blur parameters of motion deblurring algorithm for single image restoration based on the point spread function (PSF) in frequency spectrum. We then introduce a modification to the Radon transform in the blur angle estimation scheme with our proposed difference value vs angle curve. Subsequently, the auto-correlation matrix is employed to estimate the blur angle by measuring the distance between the conjugated-correlated troughs. Finally, we evaluate the accuracy, robustness and time efficiency of our proposed method with the existing algorithms on the public benchmarks and the natural real motion blurred images. The experimental results demonstrate that the proposed PSF estimation scheme not only could obtain a higher accuracy for the blur angle and blur length, but also demonstrate stronger robustness and higher time efficiency under different circumstances.

## 1 Introduction

Motion blurring is generated inevitably by camera shake during exposure time. As one of the main causes of image degradation, it seriously affects the performances of computer vision system in various fields. So efficient motion deblurring technology is conducive to improving the reliability of related applications, such as aerospace, medical imaging, traffic monitoring, public safety, military search, satellite and space image [1–3].

Some of the latest methods adopt deep learning to predict the probabilistic distribution of motion blur and recover the degraded images [4–10]. These deep learning approaches can be classified into two categories. The first kinds of methods rely on multi-frame images, and they own a complex network structure, so they are time-consuming [7, 9, 10]; the second types of these approaches only use single image to deblur the degraded image, this kind of method is simple in network structure and fast in training [4, 5, 11], but they still have some shortcomings: Aizenberg developed a multi-layer neural network (MLMVN) [4] to conduct blur identification, however, MLMVN is concentrated almost on horizontal blur [5]. Dash has developed a Gabor filter and radial basis function neural network (RBFNN) [5] to estimate the blur parameters in frequency response. Even though Gabor filter and RBFNN work well on the estimation of PSF parameters, they require sufficient Gabor filter masks in various orientations to ensure its accuracy [12]. Two kinds of deep learning methods are either time-consuming to calculate the complex training structure or have special requirements for blur conditions, both of them are not suitable for single image deblurring. In addition, some researchers have proposed efficient infrastructures to increase the operational efficiency of image processing applications, e.g. partial differential equation (PDE), multi-sink distributed power control algorithm (MSDPC-SRMS), wireless sensor networks, wireless mesh networks (WMNs), hidden Markov model (HMM) and ALOHA protocol [13–18]. However, these efficient infrastructures are not suitable to the application of image deblurring, since the research theory of image restoration is different from other image processing areas. A popular way to tackle motion deblurring of single image is to deconvolute the blurred image with PSF [19].

Intuitively, the motion blurred image can be usually modeled as a convolution of the original image with PSF, which can also be named blur kernel. In general, the motion blurred image restoration techniques are used to eliminate or minimize the impact of PSF from the degraded image. Early research on motion deblurring are mostly focusing on proposing effective algorithm to inverse the process of image degradation, i.e., deconvolution of the blurred image. These researches can be roughly divided into two categories: blind and non-blind deblurring. The non-blind deconvolution methods are put forwards to conduct image restoration by a predetermined PSF, it is assumed that PSF is already known, such as the non-iterative Wiener filter [20], Iterative Lucy-Richardson algorithm [21], Bayesian deconvolution [22], and their corresponding improved methods etc. These well-known algorithms are widely adopted in deblurring, since most of them could restore the blurred image. The core issues of PSF are the two motion parameters: angle and length of motion. In the real world circumstances, due to the unknown motion information of camera and object, the values of blur angle and length are always unavailable.

Blind image deconvolution is an improved solution for motion blur image restoration, which firstly estimates two motion parameters from the blurred image and then restores the original appearance through the detected PSF. This kind of methods is clearly harder than non-blind counterpart and far more significant in estimating PSF, thus many researchers are focus on it [4, 23–28]. In order to result in an efficient iterative process, Figueiredo et al. proposed a method based on the fast Fourier transform (FFT) and the discrete wavelet transform (DWT) to conduct the image restoration [24]. Aizenberg et al. proposed a multi-layer neural network based on multi-valued neurons (MLMVN) to identify both types and parameters of the PSF [4]. Besides, Hong et al. proposed an adaptive PSF estimation method based on an-isotropic regularization to improve the precision of the blur kernels, whose method adopts the estimated blur kernel and the proposed maximum likelihood (ML) estimation deblurring [26]. Most of these algorithms have been proved to be able to estimate very complex blur kernels accurately and yield impressive results. However, in practical applications, these methods are complicated in computational framework and time-consuming, since there are often numerous equations needed to be solved in the calculation processes.

**Fig 1:**
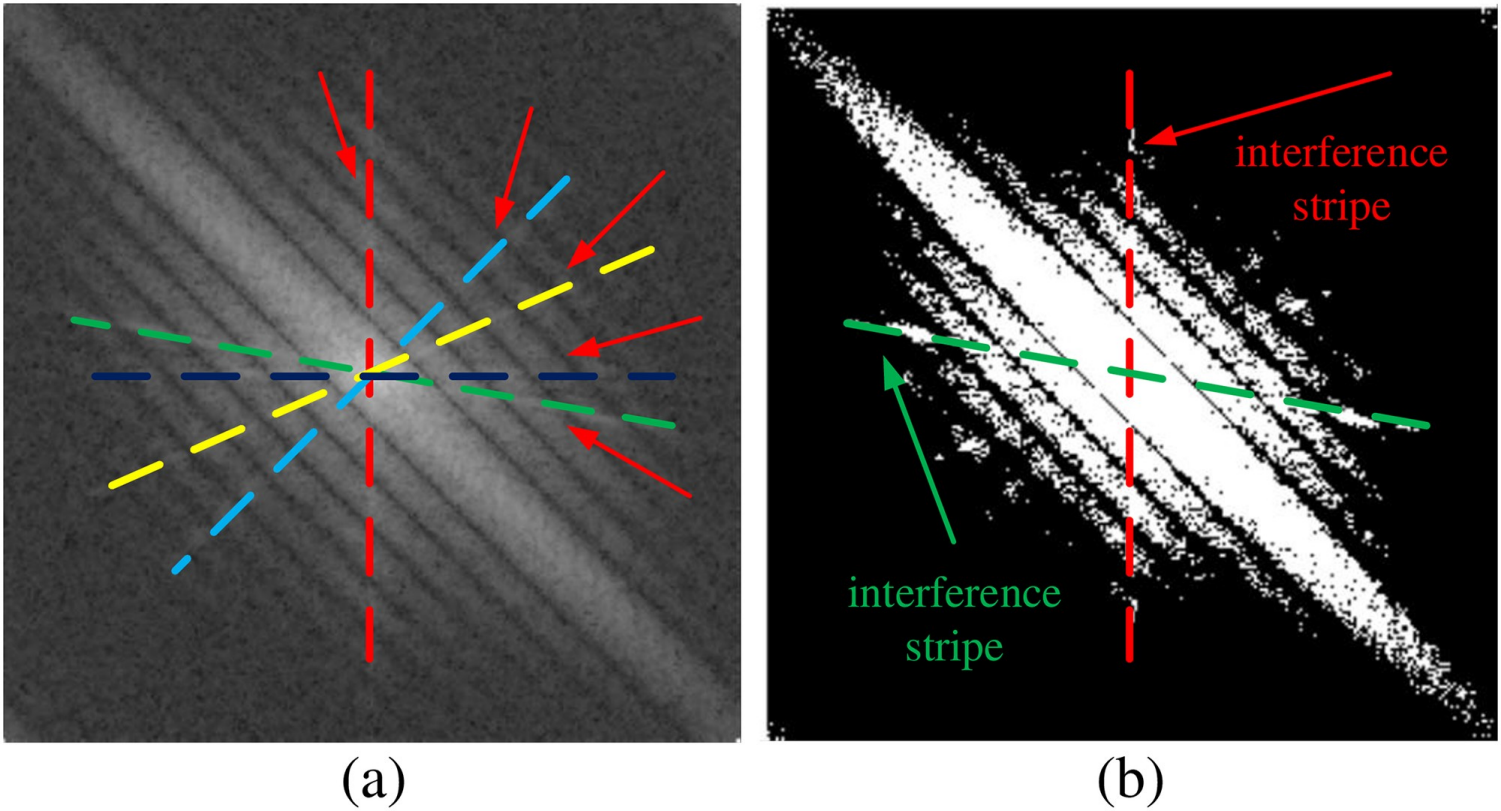
The interference stripes of image. (a) The interference stripes in the original spectrum image; (b) The interference stripes after the adaptive median filter and binarization on the spectrum image.

Beyond that, some researchers have proposed some other PSF calculation algorithms that focus on estimating the blur angle and length for blur image restoration. Oliveira and Figueiredo et al. proposed a spectrum-based method to estimate the parameters of two types of blurs (linear-uniform and out-of-focus motions) for blind image restoration [29]. In their method they introduce two modifications to the Radon transform [30] to estimate the blur parameters, and the effectiveness of their proposed method is verified on the real natural blurred images. Sun and Cho et al. introduced a patch-based strategy for blur kernel estimation, their method estimates a “trusted” subset of *x* by imposing a patch prior specifically tailored towards modeling the appearance of image edge and corner primitives [31]. Deshpande and Patnaik proposed a modified cepstrum domain approach combined with bit-plane slicing method to estimate uniform motion blur parameters [32]. Cho et al. incorporated the Radon transform within the maximum a posteriori (MAP) estimation framework to jointly estimate the blur kernel and deblur the image, this algorithm performs well on a broader variety of scenes [25]. Aiming at improving the robustness for noisy images, Moghaddam and Jamzad combined the Radon transform and bi-spectrum modeling to quantify the motion blur parameters [33]. However, the restoration accuracy of this method is weak, especially for the estimation of large blur length. Wang et al. introduced an improved PSF parameters estimation algorithm which combined bilateral-piecewise estimation strategy and the sub-pixel level image generated with bilinear interpolation in different noisy situations [12]. However, it is too hard for this algorithm to explore effective solution under non-linear and non-uniform motion blur. The Hough-transformation-based method estimates the PSF parameters by means of the log spectrum of the blurred images [34]. This method owns an obvious shortcoming that is the choosing of threshold values during image binarization, and therefore the error in angle estimation will result in erroneous length estimation [12]. Most of the methods are still not able to produce good precision results especially the spectrum image containing interference stripes (see the colored lines of dashes in Fig 1), and large blur length estimation. On the whole, the existing algorithms still can not achieve a satisfactory balance between precision, robustness and time efficiency.

**Fig 2:**
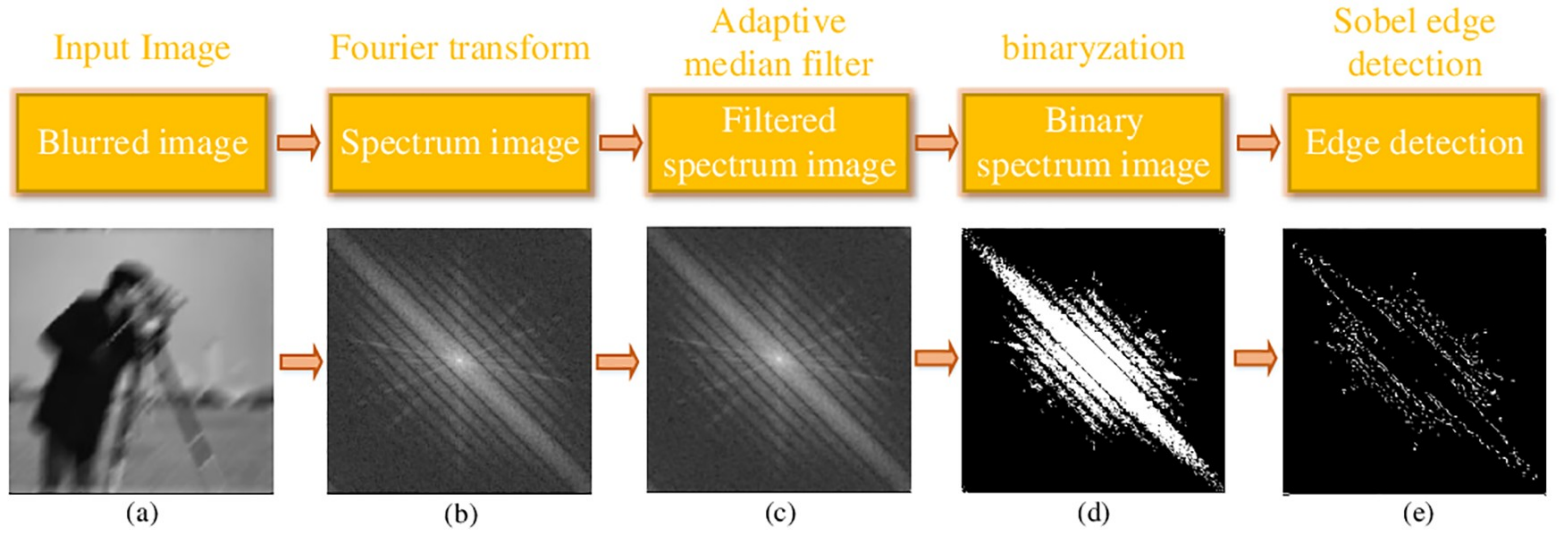
Spectrum image processes. (a) Inputting blurred image; (b) Obtaining spectrum image; (c) Filtering spectrum image; (d) Conducting binarization of the spectrum image; (e) Carrying on Sobel edge detection.

To address these limitations in single blind image restoration, we propose a novel Radon-transform-based blur angle estimation scheme which is inspired by the dark and bright stripes in frequency domain (see also in Fig 2). Based on this blur angle estimation method we also propose an accurate auto-correlation-matrix-based approach to detect the blur length. Comparing with the traditional stripe-based estimation method, the underlying techniques used in this paper are different and more advanced.

- We firstly design and conduct a set of preprocessing to filter the original spectrum image by the adaptive median filter [35], binarization and Sobel edge detection etc.
- Secondly, differently from the previous Radon-transform-based methods [25, 36, 37], we not only detect the blur angle but also refine it by our proposed tri-Radon transform method.
- Thirdly, in the second and third Radon transform, we constructed a Difference value vs Angle curve to estimate the angle.
- Fourthly, during the blur angle estimation, our own designed minimum-based filtering algorithm is employed to decrease the influence from the interference stripes. Besides, the adaptive median filter, binarization and Sobel edge detection in spectrum image preprocessing are also benefited to weaken the affection from interference stripes.
- Fifthly, we combine the first order differential of image and the auto-correlation matrix to estimate the blur length accurately and efficiently.
- In addition to the above, our proposed method can deal with the image of arbitrary sizes of row and column, since the method proposed in [12] can only handle the square image.

## 2 General motion blur model

The degradation process of blurred image can be approximately simulated as a liner degradation system, and the blurred image caused by the relative motion between camera and scene can be described as a two-dimensional convolution model in the linear space translation, which is modeled as:

$$\begin{array}{c} g(x,y)=f(x,y)*h(x,y)+n(x,y), \end{array}$$ (1)

where *g*(*x*, *y*),*f*(*x*, *y*) represent the blurred and original image respectively, *h*(*x*, *y*) is the PSF function or blur kernel, and *n*(*x*, *y*) refers to the additive noise. The notation “*” represents the convolution operator.

From Eq (1), we can see that the keypoint of deblurring is to determine the PSF function *g*(*x*, *y*). Assuming that the scene objects move uniformly relative to the camera, we can deduce that the gray values of any points in the blurred image are related to the gray values of their corresponding adjacent points in the original image, and the PSF for motion blurring can be expressed as [12]:

$$\begin{array}{c} h\left(x,y\right)=\{\begin{array}{c} 1/l,0\leq |x|\leq l\cos(\theta ),|y|=l\sin(\theta )\\ 0,otherwise \end{array} \end{array}$$ (2)

where *l* is the length of blur, and *θ* represents the angle of blur.

## 3 Blur angle estimation

As mentioned previously, the blur angle can be obtained by measuring the direction of the approximately linear dark stripes in frequency spectrum. In this section, we adopt a modified Radon-transform-based method to detect the blur angle. The application of Radon transform in image processing can be expressed as:

$$\begin{array}{c} \begin{array}{c} R_{\theta }(x^{\prime })=\int_{-\infty }^{+\infty }f(x^{\prime }\cos\theta -y^{\prime }\sin\theta ,x^{\prime }\sin\theta -y^{\prime }\cos\theta )dy^{\prime }\\ \left(\begin{array}{c} x^{\prime }\\ y^{\prime } \end{array})=(\begin{array}{cc} \cos\theta & \sin\theta \\ -\sin\theta & \cos\theta \end{array})(\begin{array}{c} x\\ y \end{array}\right) \end{array} \end{array}$$ (3)

where *R*_*θ*_(*x*′) is the value of Radon transform projection, *θ* represents the angle of Radon transform, (*x*, *y*) and (*x*′, *y*′) refer to the coordinates of original image and Radon transformed image respectively.

Radon transform is regularly used to detect the lines in the image. There are several dark stripes tilted at a certain angle in the spectrum figure of motion blurred image, and these dark stripes are parallel and symmetrical. Normally, the blur angle can be obtained by detecting the peak value with Radon transform for these dark stripes in the spectrum image. Most of the Radon-transform-based methods do not deal with the spectrum image before using radon transform to detect the blur angle. In practical application, the light and dark stripes in spectrum image are often ambiguous, especially the blur image with noise, this will lead to large errors in the estimation of blur angle, and thus will affect the detection of blur length and the deblurring of degraded image in the following phases of the whole algorithm. To overcome this problem, our blur angle estimation is based on the diversification processing of spectrum image.

### 3.1 The spectrum processing

The outline of our spectrum image processing is presented in Fig 2. Here, we take the cameraman.tif with a blur angle of 45° as a sample in the processes. As is shown in Fig 2(b), before our method starts, the spectrum image is obtained by Fourier transform of blurred image.

Due to the influence from noise, camera and other image acquisition equipment, there are many interference stripes in the spectrum image, such as the lines of dashes marked wit red, sky-blue, yellow, dark-blue and green colors in Fig 1(a) and 1(b). To eliminate the disturbances of interference stripes, we adopt adaptive median filter, binaryzation and minimum-based filtering algorithm. The adaptive median filter and binaryzation are carried out on the spectrum image in this section, and the details of minimum-based filtering algorithm are presented in the Section 3.2. According to the above analysis, we then use the adaptive median filter on the spectrum image, so as to eliminate the interference from the noise (see also Fig 2(c)). Following the experiments experience, the appropriate size of adaptive median filter should be 3. After that, in the process of Fig 2(d), we binarize the filtered spectrum image with a threshold *τ*_*b*_.

When we get the binarization result for the filtered spectrum image, we consider whether to conduct the edge detection for the spectrum image by a threshold *τ*_*e*_, and the Sobel operator is adopted in the edge detection algorithm (see also in Fig 2(e)). Based on the whole experiments experience, the suitable value for threshold *τ*_*b*_ and *τ*_*e*_ should be 0.35 and 0.47.

Then the Radon transform is made on the binarization spectrum image to detect the blur angle.

### 3.2 Tri-Radon transforms

When the binary spectrum image is obtained, the next step is to estimate the blur angle by Radon transform. In the whole algorithm, we totally adopt Radon transform projection three times to detect the blur angle, which we named “Tri-Radon” transforms.

**Fig 3:**
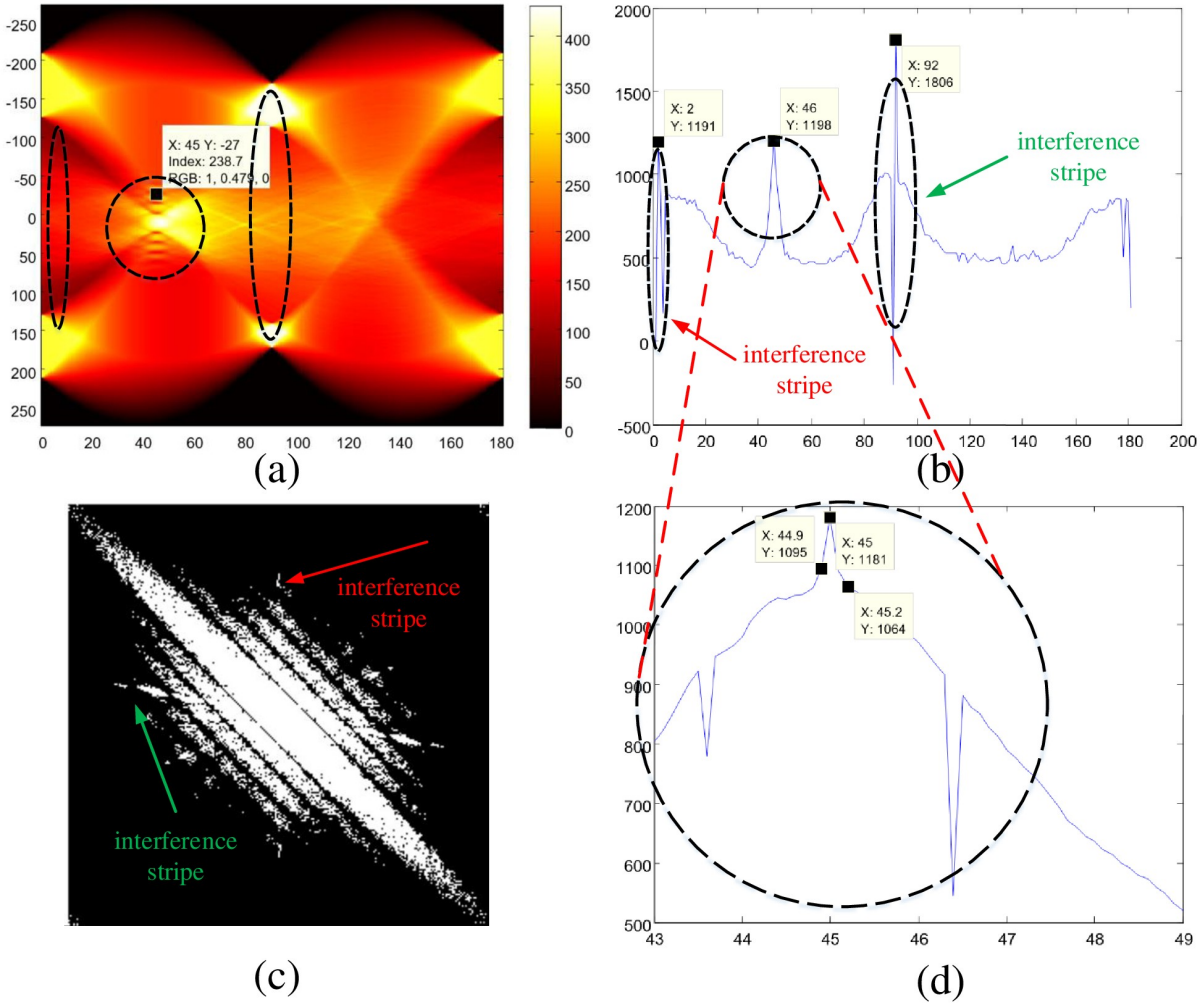
Blur angle detection by double Radon transforms. (a) The two-dimensional color map of Radon transform projection for spectrum image (first Radon transform); (b) The second rough detection of blur angle (second Radon transform); (c) The affected binarization spectrum with interference stripes in the directions of 2° and 92°. (d) The final precise detection of blur angle (third Radon transform).

In the first Radon transform process, we firstly detect the blur angle by the two-dimensional color map of Radon transform projection roughly (see also in Fig 3(a)). In the color map of Radon transform projection, the horizontal coordinates *θ* (from 0° to 180°) represent the angle of Radon transform projection, the vertical coordinates *x*′ (from -250 to 250) refer to the distance from a straight line to the origin in the binaryzation spectrum, and this line is perpendicular to a straight line with a such angle (the horizontal coordinates’ value), the color bar at the right part of color map means the value of Radon transform projection *R*_*θ*_(*x*′). For better understanding, here we take the coordinates value (45°, -27, 238.7) in the color map as an example, where 45° is the value of horizontal coordinates *θ*, -27 is the value of vertical coordinates *x*′, and 238.7 is the value of Radon transform projection *R*_*θ*_(*x*′), this means that there is a straight line passing through the origin with a slope of 45°, and there also exists a straight line *B* perpendicular to *A* and 27 distances away from the origin in the binarization spectrum, since the value of Radon transform projection at this position is 238.7. When we comprehend the meaning of Radon transform projection’s color map, we can observe that there are many local maximum and minimum values of Radon transform projection on straight lines with a slope of 45° in Fig 3(a), especially the neighborhood of origin (vertical coordinate 0) in spectrum image. From this observation, it can be judged that the blur angle *θ*_*psf*_ is about *θ*_1_ = 45°:

$$\begin{array}{c} \theta_{psf}=\theta_{1} \end{array}$$ (4)

After the rough estimation of blur angle by the two-dimensional color map of Radon transform projection for spectrum image, the next step is to conduct the blur angle detection through the second Radon transform.

As is shown in Fig 3(b), in the second Radon transform projection for the binary spectrum image, we set the angles *θ*_2*p*_ of projection from 0° to 180° and step width to 1°. After the value of Radon transform projection for each angle is obtained, we firstly calculate the summations of local maximums and local minimums respectively, then we calculate the difference between these two summations:

$$\begin{array}{c} \begin{array}{c} D_{\theta }=\sum_{1}^{m}\left(Max_{\theta 1}+Max_{\theta 2}+\cdots +Max_{\theta m}\right)\\ -\sum_{1}^{n}\left(Min_{\theta 1}+Min_{\theta 2}+\cdots +Min_{\theta n}\right) \end{array} \end{array}$$ (5)

where *Max*_*θm*_ and *Min*_*θn*_ represent the local maximum of Radon transform projection on spectrum image with angle *θ*, and *D*_*θ*_ is the difference between the summation of local maximums and the summation of local minimums. Based on the data of *D*_*θ*_ and *θ*, the Difference value vs Angle curve can be obtained (see also in Fig 3(b) and 3(d)). Through this curve, we can obtain the rough estimation of blur angle *θ*_2_ by searching the corresponding angle of the projection value’s local maximum:

$$\begin{array}{c} \forall |\theta_{2p}-\theta_{2}|\leq 10,\exists D_{\theta_{2}}>D_{\theta_{2p}}\Rightarrow \theta_{psf}=\theta_{2} \end{array}$$ (6)

Through the experiments experience, we can find that in all the Difference value vs Angle curves, there are two additional angles (2°, 92°) detected except the true blur angle (46°), since the binarization spectrum is affected by the two tiny stripes in the direction of 2° (red arrow marked) and 92° (green arrow tagged) in Fig 3(c). Through all experiments’ Difference value vs Angle curves, we can observe that there is a minimum in the neighborhood angle of false positive (FP) results:

$$\begin{array}{c} \begin{array}{cc} & \exists \theta_{i}\in \left[\theta_{2}-5,\theta_{2}+5\right],\forall \left|\theta_{j}-\theta_{i}\right|\leq 5,\exists D_{\theta_{i}}<D_{\theta_{j}}\\ & \Rightarrow \theta_{psf}\neq \theta_{2} \end{array} \end{array}$$ (7)

So, to eliminate the interference from the noisy stripes, we adopt our proposed minimum-based filtering algorithm as following:

1. Firstly, we’ll search more than three largest maximums of *D*_*θ*_ in the Difference value vs Angle curves of angle from 0° to 180°;
2. After that, we will judge whether there is a minimum in the neighborhood angle of each maximum value’s corresponding angle, then we delete the elements with minimum values;
3. The remaining maximum’s corresponding angle identified as the blur angle *θ*_2_ of second estimation.

Based on the second rough blur angle estimation results, we conduct the third Radon transform projections for the binary spectrum image. This time the angles of projection are set from *θ*_2_ − 3° to *θ*_2_ + 3°, and the step width is set to 0.5° (see also Fig 3(c)). Then we repeat the steps of the second blur angle estimation. Through the Projection value vs angle curve of the third Radon transform projections, we can get the precise blur angle estimation results *θ*_3_, and we update *θ*_3_ as the ultimate blur angle:

$$\begin{array}{c} \forall |\theta_{3q}-\theta_{3}|\leq 2,\exists D_{\theta_{3}}>D_{\theta_{3p}}\Rightarrow \theta_{psf}=\theta_{3} \end{array}$$ (8)

All the above estimations of blur angle are for square images, if the sizes of image’s row and column are not consistent, the blur angle can obtained as following:

$$\begin{array}{c} tg\theta =-ctg\Theta \cdot \frac{N}{M}, \end{array}$$ (9)

where Θ is the angle estimation from the rectangle image, *θ* represents the blur angle, *M* and *N* refer to the sizes of image’s row and column respectively.

## 4 Blur length estimation

**Fig 4:**
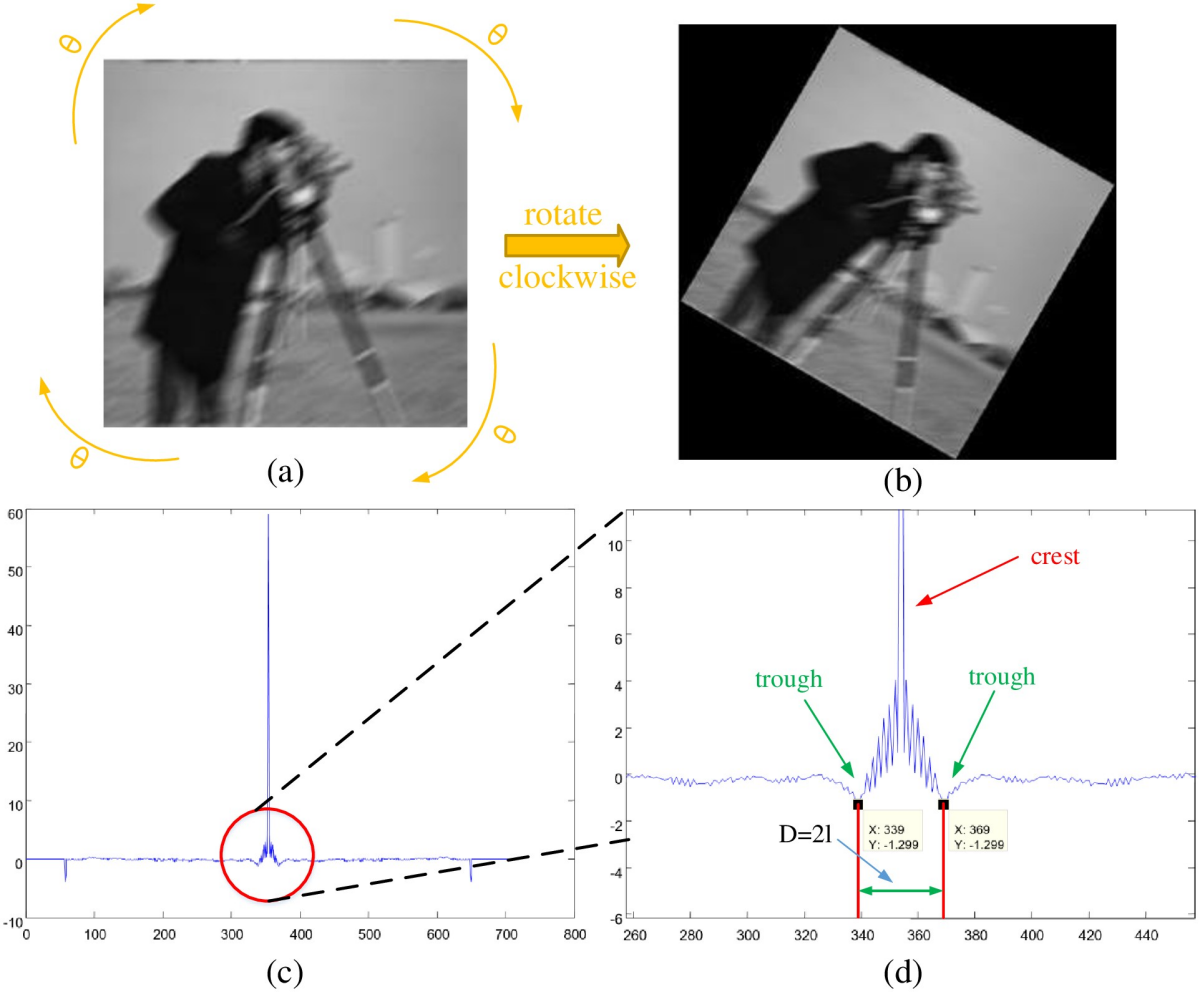
Blur length estimation processes. (a) The original blurred image; (b) The rotated blurred image *Z*(*x*, *y*); (c) The *S*′(*y*) vs *y* curve; (d) The enlarged version of *S*′(*y*) vs *y* curve.

The precondition of our blur length estimation is to firstly measure the blur angle and then rotate the blurred image clockwise with the detected blur angle (see also in Fig 4(a)), thus the motion becomes in horizontal direction, and we can obtain the rotated blurred image *Z*(*x*, *y*) in Fig 4(b). Firstly, the first order differential of image *Z*(*x*, *y*) in horizontal direction is given by:

$$\begin{array}{c} Z^{\prime }\left(x,y\right)=\frac{dZ(x,y)}{dx}, \end{array}$$ (10)

where *Z*′(*x*, *y*) is the first order differential of *Z*(*x*, *y*). Then we calculate the auto-correlation matrix of *Z*′(*x*, *y*) in horizontal direction as follows:

$$\begin{array}{c} S\left(x,y\right)=\sum_{p=0}^{M-1}Z^{\prime }\left(x,p\right)Z^{\prime }\left(x,p+y\right) \end{array}$$ (11)

where *M* is the size of *Z*′(*x*, *y*)’s row, which equals to the size of image *Z*(*x*, *y*)’s row, and *S*(*x*, *y*) refers to the auto-correlation matrix of *Z*′(*x*, *y*) in horizontal direction. According to the auto-correlation matrix *S*(*x*, *y*), we can calculate the summations of each column elements in *S*(*x*, *y*):

$$\begin{array}{c} S^{\prime }\left(y\right)=\sum_{x=0}^{M-1}S\left(x,y\right) \end{array}$$ (12)

where the size of *S*′(*y*) is 1 × *N*. The goal of *S*′(*y*)’s calculation is to reduce the influence from noise and improve the accuracy of blur length estimation. The blur length can be estimated through the *S*′(*y*) vs *y* curve in Fig 4(c) and 4(d), where the horizontal coordinate is the data of *y*, and the vertical coordinate is the corresponding *S*′(*y*). We need to search a pair of conjugated-correlated troughs (tagged with green arrows) on the right and left sides of central crest (marked with red arrow) in the *S*′(*y*) vs *y* curve, the half *l* of the distance *D* between these two conjugated-correlated troughs is the estimated blur length. Here we take an experiment result as the sample in Fig 4(d), we can see that the horizontal values of these two conjugated-correlated troughs are 339 and 369, and the distance *D* between these two troughs is 30, so we can estimate the blur length is $\frac{1}{2}*30=15$. In summary, our blur length estimation is proposed as follows:

1. We rotate the blurred image *g*(*x*, *y*) clockwise to the horizontal motion direction by the detected blur angle, thus we can obtain the rotated blurred image *Z*(*x*, *y*).
2. Calculating the first order differential *Z*′(*x*, *y*) of *Z*(*x*, *y*) in horizontal direction.
3. Then calculating the auto-correlation matrix *S*(*x*, *y*) of *Z*′(*x*, *y*).
4. Computing the summations of each column elements in *S*(*x*, *y*) to get *S*′(*y*).
5. Estimating the blur length by searching a pair of conjugated-correlated troughs in the *S*′(*y*) vs *y* curve.

With the blur parameters estimation algorithms mentioned in Section 3 and Section 4, we can construct the PSF function by Eq 2. Then we can restore the blurred image by the non-blind filter method.

## 5 Experiments

In this section we conduct a series of comparisons between our approach and the state-of-the-art methods (Moghaddam [33], traditional Radon-transform-based method [25], deep learning approach (RBFNN) [5] and Wang [12] are carried out in this section). A total of 18625 images including VOC2012 dataset [38] and real-life images are used to evaluate our approach in Section 5.1, 5.2 and 5.3.

### 5.1 Performance of the parameters estimation approaches

In this subsection, we evaluate our proposed parameters estimation approaches by the degraded images with various blur angles and lengths, and the blurred images which are generated by varying the blur angle from 0° to 180° and the blur length from 0 to 100 pixels respectively.

First of all, the absolute errors for two blur parameter estimations using the five methods are shown in Table 1. From this table, we can get several observations:

1. During the whole experiments, all the methods tend to almost no error for min absolute errors of blur angle *θ* and blur length *L*.
2. No matter which cases, Wang’s approach and our proposed method are obviously superior to the first three methods. Moghaddam’s approach obtains the worst performances for the max absolute errors of *θ* and *L* among the five methods, but it could produce comparative mean absolute error with traditional Radon-based and Dash’s methods for *θ*.
3. In sharp contrast, our method achieves the best estimation accuracy for both *θ* and *L* with the least mean absolute errors of 0.11° and 0.14 pixels. Moreover, the max absolute errors of these two parameters generated by our approach are less than 0.22° and 0.28 pixels respectively, significantly smaller than those generate by the other four approaches.

**Table 1:** Estimation accuracy comparison between five methods for two blur parameters.

Method	Moghaddam [33]		Traditional Radon [25]		RBFNN [5]		Wang [12]		Our method	
Para	*θ*(°)	L (pixel)	*θ*(°)	L (pixel)	*θ*(°)	L (pixel)	*θ*(°)	L (pixel)	*θ*(°)	L (pixel)
Min.absolute error	0	0	0	0	0	0	0	0	**0**	**0**
Max.absolute error	2.01	2.21	0.99	1.26	0.89	1.03	0.26	0.32	**0.22**	**0.28**
Mean.absolute error	0.63	1.17	0.60	0.97	0.58	0.89	0.12	0.18	**0.11**	**0.14**

**Fig 5:**
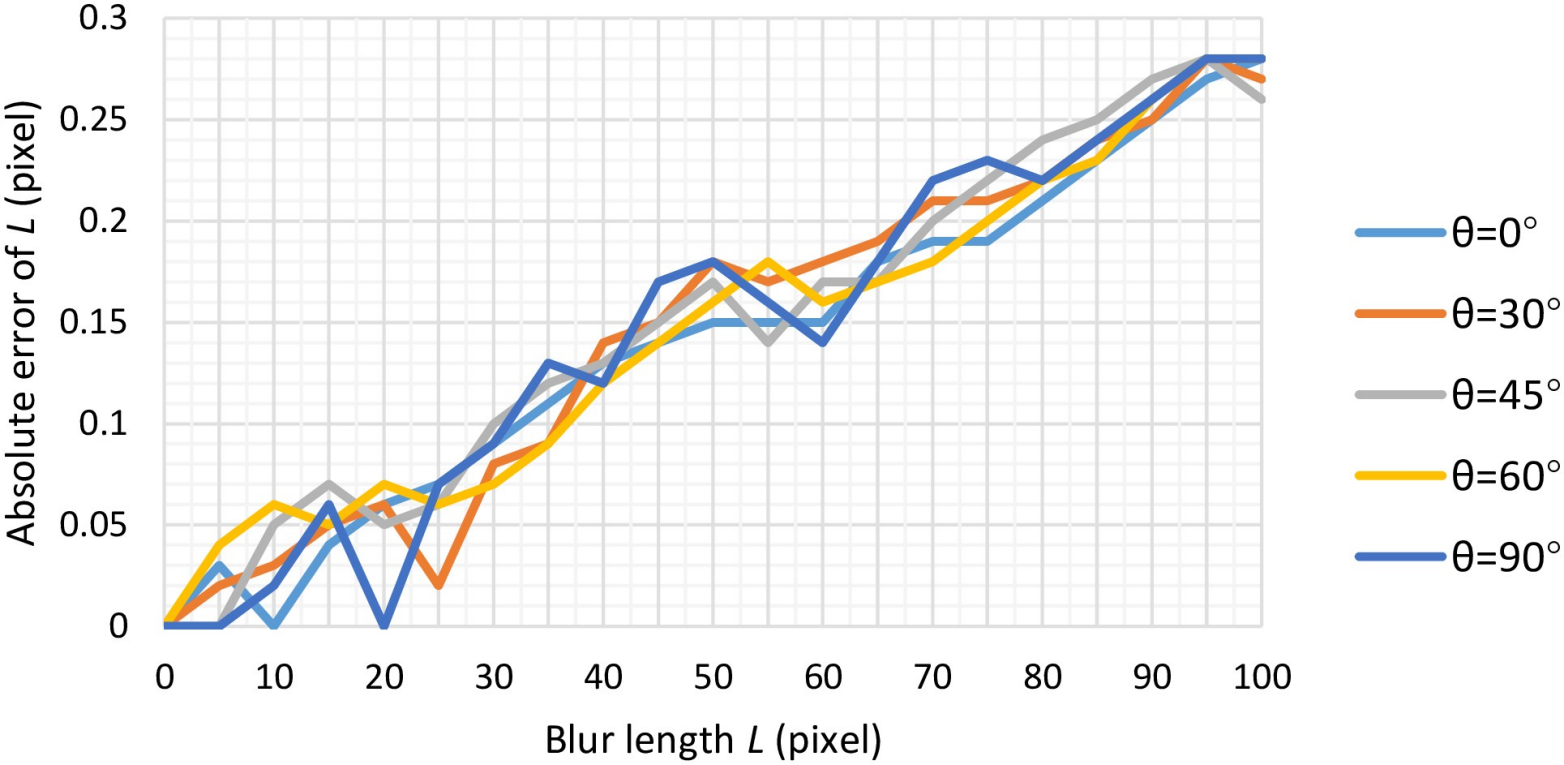
Absolute error of blur length *L* from our proposed method.

Besides, by varying *L* from 0 to 100 pixels with a step of 5 pixels at the blur angle of 0°, 30°, 45°, 60° and 90° respectively, the absolute error with respect to the blur length *L* is observed, the experiment results are shown in Fig 5. Obviously, the absolute error curve fluctuates greatly with a lower value when *L* is small, and then the error increases slowly when *L* is larger than 30 pixels. This can be owing to the fact that the interference stripes are generated in the spectrum image when the blur length is large (see also in Figs 1 and 3(c)). Under these circumstances, it may lead to the increasing estimation error of the distance between a pair of conjugated-correlated troughs in Fig 4. Furthermore, our proposed blur length estimation method achieves the best performance of near 0 pixels absolute error with *L* = 0 pixels & *θ* = {0°, 30°, 45°, 60°, 90°}, *L* = 5 pixels & *θ* = {45°, 90°}, *L* = 10 pixels & *θ* = 0°, *L* = 20 pixels & *θ* = 90°. Under any instances, the absolute error of *L* is less than 0.28 pixels no matter how *L* and *θ* varies in the range (0 ≤ *L* ≤ 100, *θ* = {0°, 30°, 45°, 60°, 90°}), which shows that the performance is very good.

**Fig 6:**
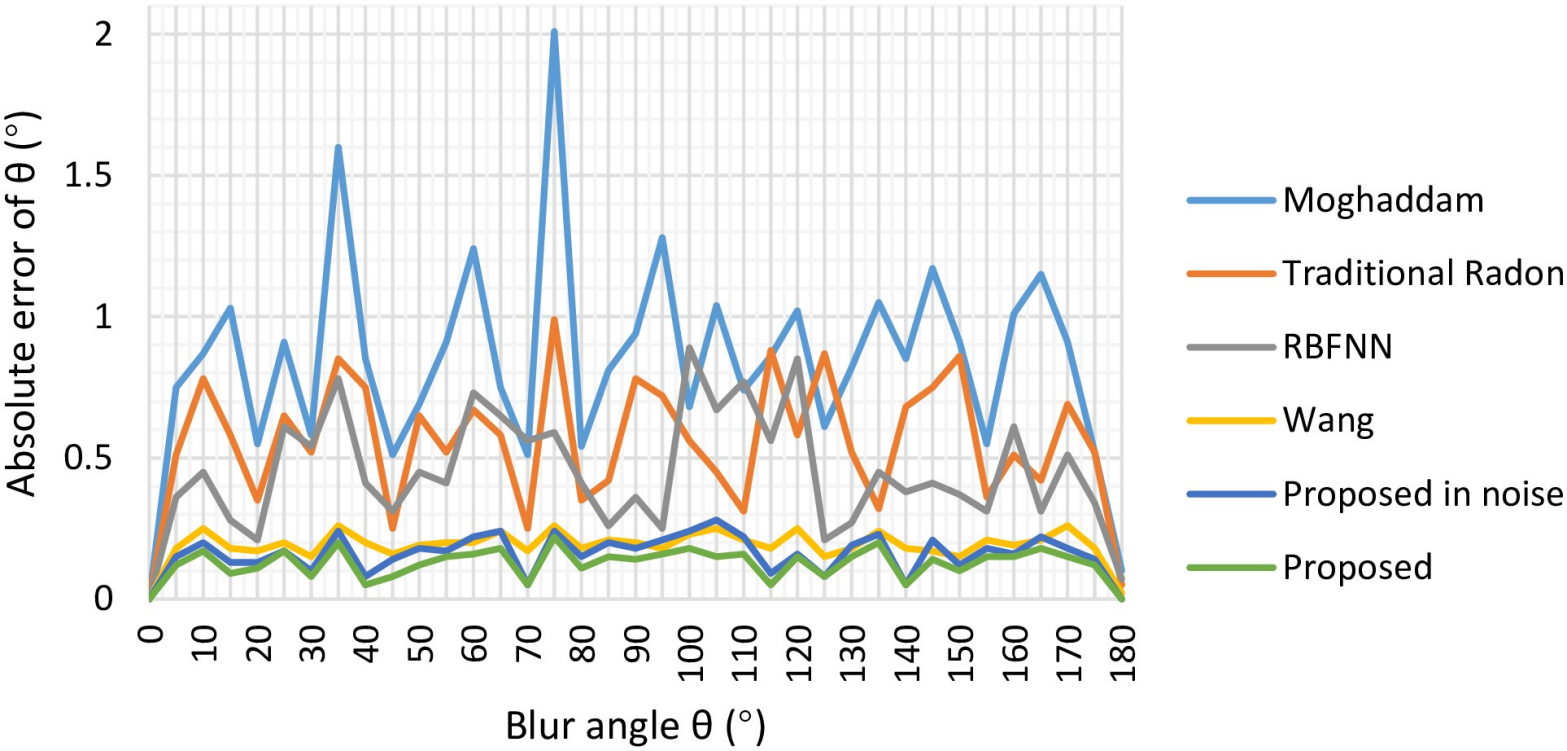
Absolute error comparison of blur angle *θ*.

**Fig 7:**
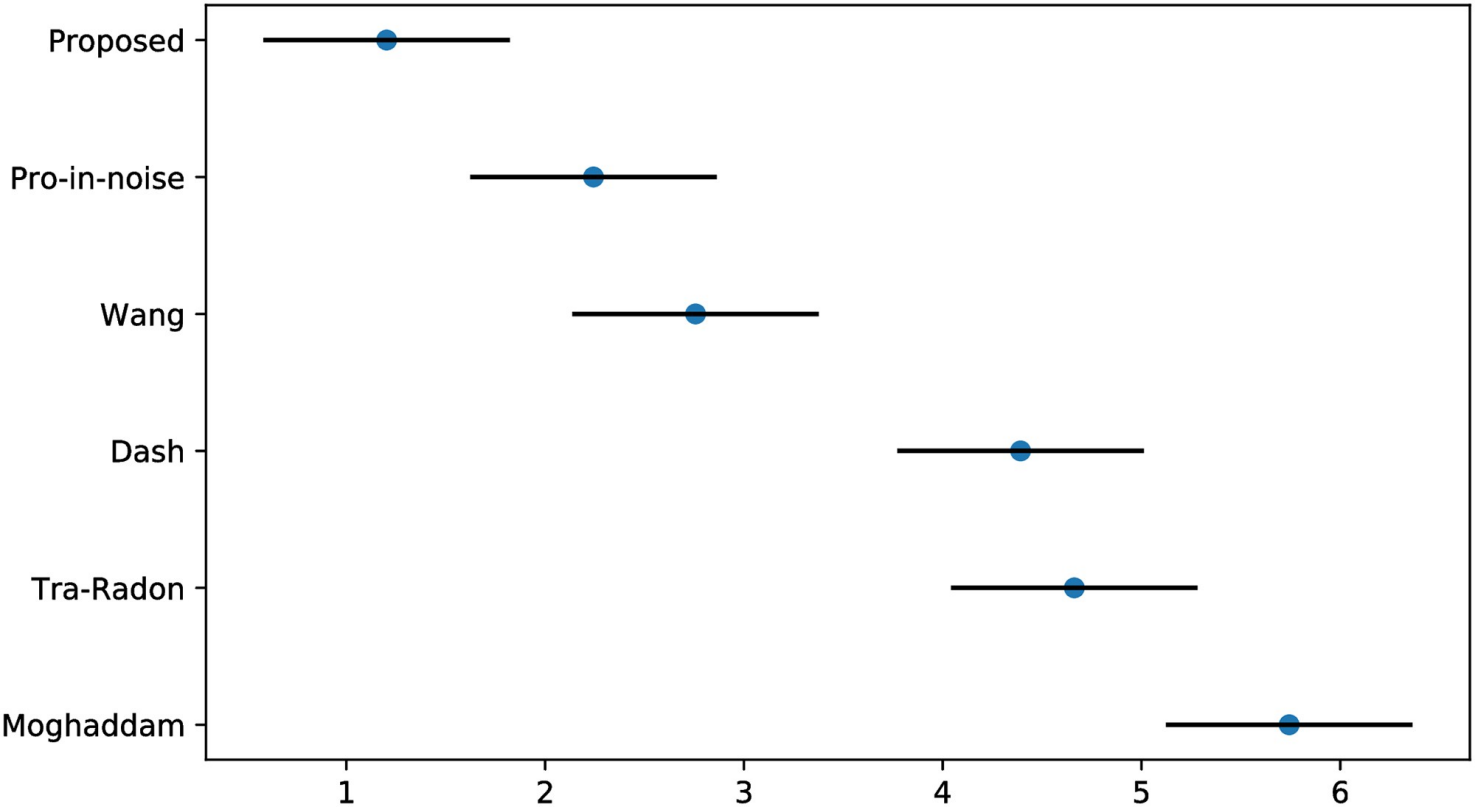
Friedman and Nemenyi tests of blur angle *θ*’s absolute errors.

Similar to the experiment for blur length *L*, the absolute error in terms of the blur angle is observed. Moghhadam’s [33], Traditional-Radon-based [25], Dash’s [5] and Wang’s [12] methods are adopted in blur angle estimation experiments to evaluate and validate the superiority of our proposed method. Meanwhile, the Gaussian noise with standard deviation *σ* = 0.0001 is also added into the degraded images to demonstrate the good performance of our method. The experiment results are presented in Fig 6. From Fig 6, we can find that our approach achieves the best accuracy results among the five methods. Besides, we also conduct additional Friedman and Nemenyi tests of blur angle’s absolute errors to compare our method with these algorithms, the critical difference diagram results generated by Friedman and Nemenyi tests are presented in Fig 7. Through Fig 7, we can observe that our proposed method obtain the best results on blur angle accuracy. On the one hand, by introducing the adaptive median filter, binarization and Sobel edge detection to weaken the affection from the interference stripes in Figs 1 and 3(c). On the other hand, our own designed minimum-based filtering algorithm is employed to decrease the influence from the interference stripes. These two measures both contributed to the smaller absolute error comparing with other four methods.

All these observations confirm the high accuracy estimation for blur parameters of our proposed methods.

### 5.2 Performance of deblurring on noise-free images

The previous subsection shows that our blur parameter estimation approach obtains good results under different circumstances. In this subsection, based on the constructed PSF with the estimated *θ* and *L* from Section 5.1, we use regularized filter to deblur the degraded image, so as to demonstrate that our method could yield better performance of deblurring than state-of-the-art approaches intuitively. For this purpose, we will carry out experiments on the VOC2012 dataset [38] and real-life blurred images, and compare our method against the Moghhadam’s [33], Traditional-Radon-based [25], Dash’s [5] and Wang’s [12] methods etc.

**Fig 8:**
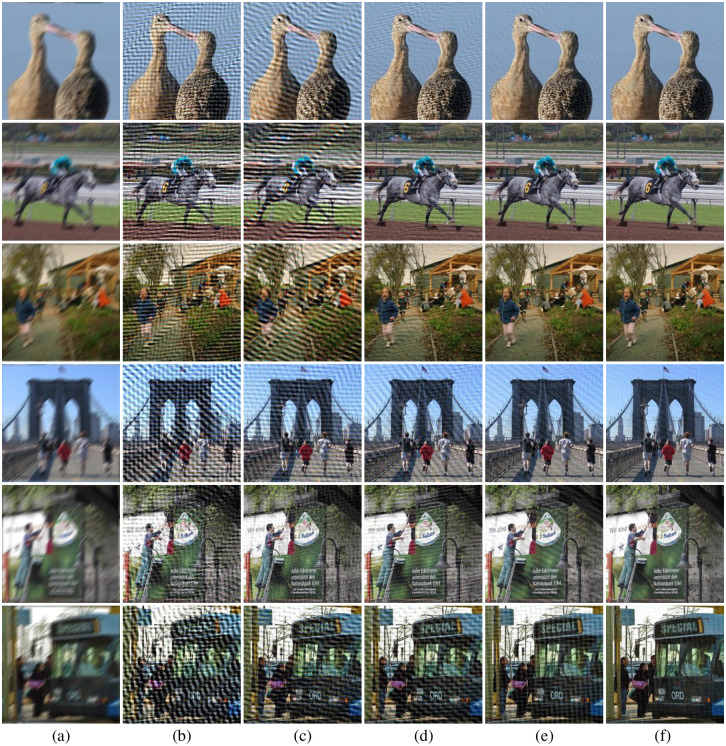
Samples of deblurring results with different methods on VOC2012 dataset. (a) degraded image, (b) Moghaddam’s results, (c) Traditional-Radon-based results, (d) RBFNN’s results, (e) Wang’s results, (f) our results.

The degraded images and restoration results produced by the five approaches in VOC2012 dataset are illustrated in Fig 8. From these figures we can observe that our method obtain the most satisfactory results and demonstrates a good robustness under different circumstances. In all the experiments, Moghaddam, Traditional-Radon-based and Dash’s methods fail to completely remove the blurring effects in the deblurred images. Wang’s approach can only obtain acceptable results in the first three rows of images.

Moreover, we adopt the peak signal-to-noise ratio (PSNR) and the running time as the evaluation criterion to quantitatively assess all methods’ performance. PSNR are defined as:

$$\begin{array}{c} \text{MSE}=\frac{1}{MN}\sum_{x=1}^{M}\sum_{y=1}^{N}{\left(f\left(x,y\right)-\hat{f}\left(x,y\right)\right)}^{2} \end{array}$$ (13)

$$\begin{array}{c} \text{PSNR}=10\log_{10}(\frac{255^{2}}{\text{MSE}}) \end{array}$$ (14)

where MSE is the mean square error, *f*(*x*, *y*) and $\hat{f}\left(x,y\right)$ are the intensity of pixel (*x*, *y*) in the image before and after motion deblurring respectively, and *M* and *N* represent the size of image. Besides, in order to make the experimental comparison more accurate, we also use structural similarity index (SSIM) [39] to evaluate the deblurring quality of each method:

$$\begin{array}{c} \mathrm{SSIM}\left(f,\hat{f}\right)=\frac{\left(2\mu_{f}\mu_{\hat{f}}+C_{1})(2\sigma_{f\hat{f}}+C_{2}\right)}{\left(\mu_{f}^{2}+\mu_{\hat{f}}^{2}+C_{1})(\sigma_{f}^{2}+\sigma_{\hat{f}}^{2}+C_{2}\right)} \end{array}$$ (15)

where *μ*_*f*_, $\mu_{\hat{f}}$, *σ*_*f*_, $\sigma_{\hat{f}}$ and $\sigma_{f\hat{f}}$ represent the local means, standard deviations and cross-covariance for images *f* and $\hat{f}$ respectively, *C*_1_ and *C*_2_ are two constants to avoid formula division by 0. Usually, the larger PSNR and SSIM, the higher restoration quality. That is, an optimal PSNR is infinity, and an ideal SSIM has value 1.

**Fig 9:**
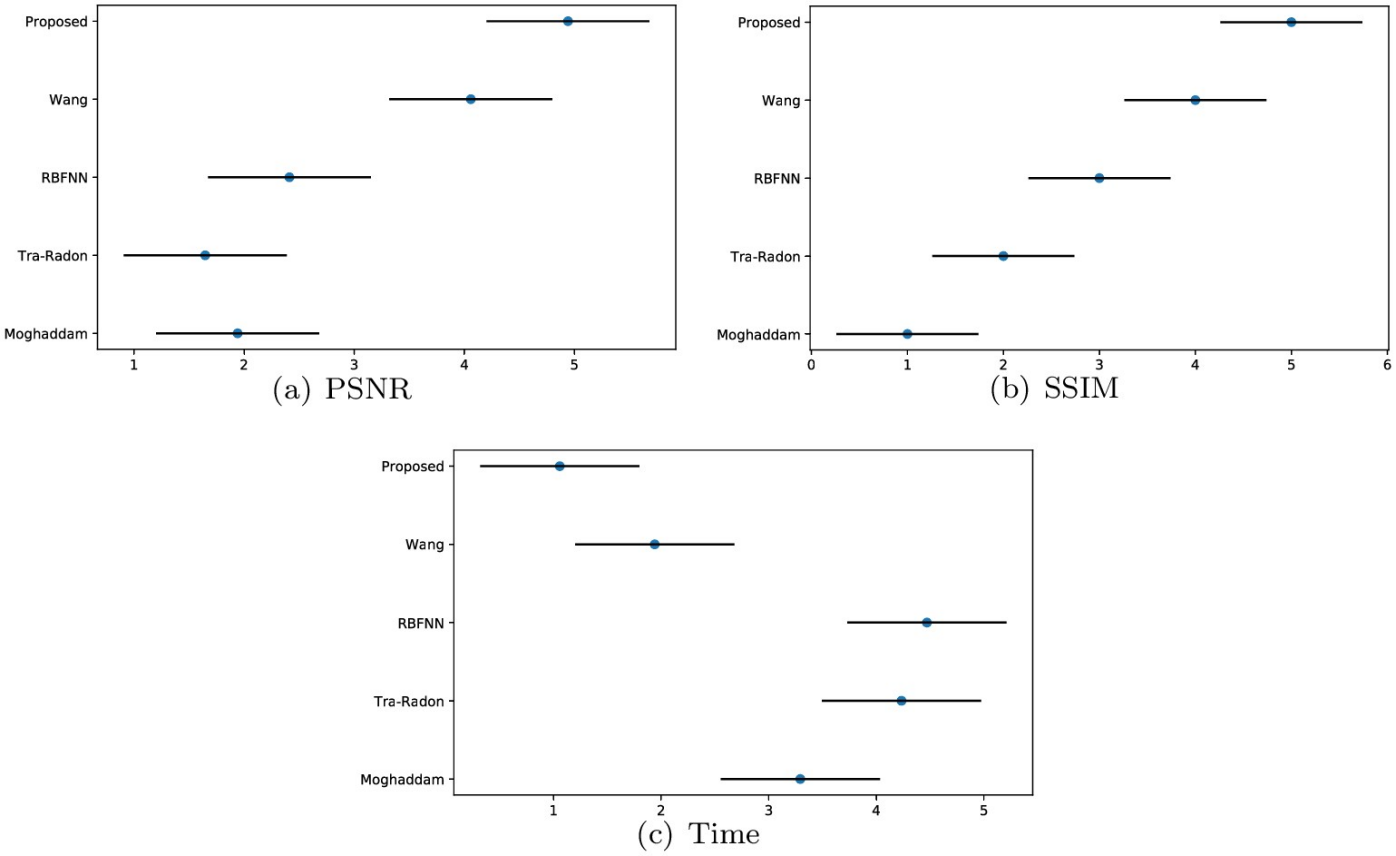
Friedman and Nemenyi tests of PSNR, SSIM and time on VOC2012 noise-free circumstances.

We experiment on the VOC2012 database to calculate each method’s PSNR, SSIM and the running time, the comparison of sample pictures and overall results are presented in Table 2. We can see clearly that our method works well on almost all sample pictures, and we obtain the largest PSNR on each image except 2008_007430.jpg, this is approximately consistent with the result in Fig 8. Wang’s approach barely has a slight advantage in obtaining better results on 2008_007430.jpg than our method. Compared with Wang and our approaches, traditional Radon-based, Moghddam’s methods and RBFNN are very time-consuming. Furthermore, our proposed method’s calculation efficiency is higher than other four approaches. In addition, we also generate the critical difference diagram results of Friedman and Nemenyi tests for PSNR, SSIM and the running time of these five methods on VOC2012 noise-free circumstance in Fig 9. It can be observed clearly that our method is quite different from other five methods, and we still obtain the best results on VOC2012 noise-free circumstances. In total, our method performs best among the five approaches, as we have the best results in 95.91% of the VOC2012 database on the image restoration quality and computing efficiency.

**Table 2:** PSNR (dB) and SSIM of the deblurred images, and the algorithms’ running time (in seconds) on VOC2012 database.

Method	Moghaddam [33]			Traditional Radon [25]			RBFNN [5]			Wang [12]			Our method		
Criterion	PSNR(dB)	SSIM	Time(s)	PSNR(dB)	SSIM	Time(s)	PSNR(dB)	SSIM	Time(s)	PSNR(dB)	SSIM	Time(s)	PSNR(dB)	SSIM	Time(s)
2007_000027.jpg	65.87	0.8214	55.34	66.38	0.9043	60.25	67.86	0.9271	53.38	78.45	0.9692	12.87	**79.04**	**0.9697**	**10.07**
2007_001733.jpg	71.79	0.9131	56.25	71.37	0.9549	59.02	70.57	0.9656	62.09	84.97	0.9858	12.80	**85.71**	**0.9861**	**10.62**
2007_005264.jpg	61.49	0.8760	48.46	61.37	0.9332	60.69	63.01	0.9484	60.55	78.47	0.9785	11.32	**79.14**	**0.9788**	**11.05**
2008_001454.jpg	63.45	0.8765	56.33	65.46	0.9299	57.65	64.18	0.9441	56.02	76.79	0.9738	11.19	**78.43**	**0.9740**	**10.33**
2008_001676.jpg	64.55	0.9220	53.52	66.71	0.9577	51.45	67.47	0.9671	63.61	84.67	0.9865	12.33	**85.57**	**0.9867**	**11.20**
2008_003974.jpg	63.23	0.9503	48.17	65.50	0.9720	59.59	66.75	0.9774	52.62	79.61	0.9890	13.71	**79.96**	**0.9892**	**10.52**
2008_004088.jpg	59.13	0.7457	49.98	57.25	0.8460	60.43	58.87	0.8756	57.47	70.45	0.9398	11.86	**71.11**	**0.9409**	**11.30**
2008_006397.jpg	62.24	0.9556	52.66	61.20	0.9724	57.88	57.08	0.9777	56.79	80.37	0.9904	12.59	**81.25**	**0.9905**	**11.37**
2008_007430.jpg	61.87	0.8778	56.77	63.64	0.9365	58.67	65.28	0.9501	61.40	**75.48**	0.9738	11.51	75.22	**0.9740**	**11.49**
2009_001117.jpg	62.99	0.6904	56.84	56.89	0.8419	58.53	60.60	0.8801	61.75	75.41	0.9447	13.09	**75.66**	**0.9454**	**10.90**
2009_003551.jpg	65.77	0.9632	48.77	63.35	0.9793	55.02	63.44	0.9836	54.45	81.17	0.9937	11.60	**81.94**	**0.9938**	**10.16**
2010_000109.jpg	63.62	0.9253	56.90	63.47	0.9620	57.65	66.71	0.9704	58.09	77.61	0.9854	12.35	**78.24**	**0.9856**	**10.45**
2011_001159.jpg	61.08	0.7765	56.77	60.19	0.8847	52.81	60.67	0.9113	57.56	76.38	0.9593	12.93	**76.89**	**0.9598**	**11.82**
2012_000279.jpg	61.44	0.6607	52.05	61.28	0.8215	58.16	62.46	0.8704	59.97	72.03	0.9436	13.51	**72.85**	**0.9444**	**10.30**
2012_002956.jpg	59.67	0.6341	55.20	65.72	0.7865	51.41	66.15	0.8334	60.72	72.77	0.9258	13.71	**72.97**	**0.9269**	**11.65**
2012_003170.jpg	62.17	0.8419	48.61	59.87	0.9136	53.86	62.66	0.9330	61.27	78.63	0.9712	12.48	**79.21**	**0.9716**	**10.15**
2012_004086.jpg	61.28	0.8643	51.41	66.18	0.9243	51.56	64.82	0.9393	55.52	74.51	0.9693	**11.25**	**76.56**	**0.9697**	11.99
*Overall*	63.14	0.8413	53.12	63.31	0.9124	57.81	63.95	0.9331	58.35	75.71	0.9577	12.48	**78.31**	**0.9711**	**10.87**

**Fig 10:**
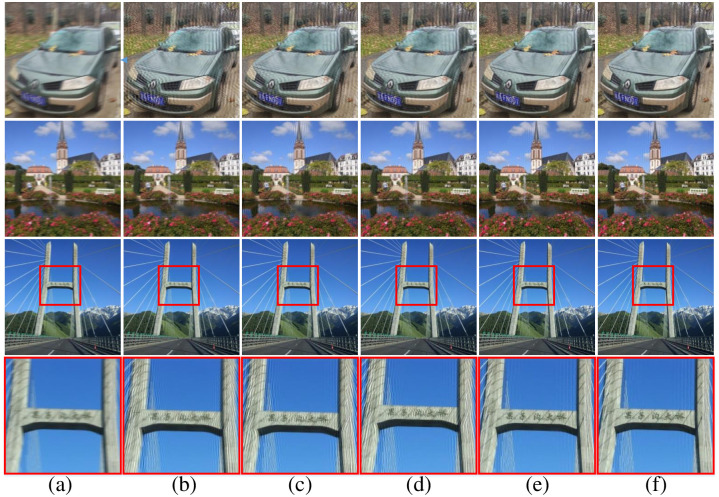
Deblurring results with different methods on real natural blurred images. (a) degraded image, (b) Moghaddam’s results, (c) Traditional-Radon-based results, (d) Dash’s results, (e) Wang’s results, (f) our results.

Additionally, we also conduct the experiments for all methods on the real captured blurred images, the experiments results are visualized in Fig 10 (car, scenery and bridge images). The car and scenery images are obtained with a hand-held camera, and the bridge image is captured in a moving car on the motorway. Based on the detection results of blur angle and blur length, the blind deconvolution is adopted to filter and deblur the degraded images. Through the Fig 10, we can also observe the similar superiority of our proposed method presented in Fig 8 and Table 2. Specifically, we can see the five Chinese characters on the horizontal beam of bridge clearly by our method through the bridge images’ enlarged version of last line in Fig 10.

### 5.3 Performance of deblurring in noisy circumstances

**Fig 11:**
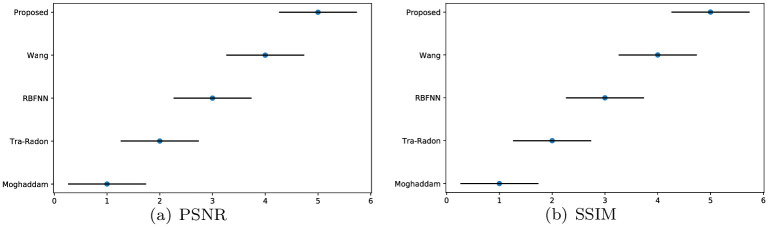
Friedman and Nemenyi tests of PSNR and SSIM on VOC2012 noise circumstances.

**Fig 12:**
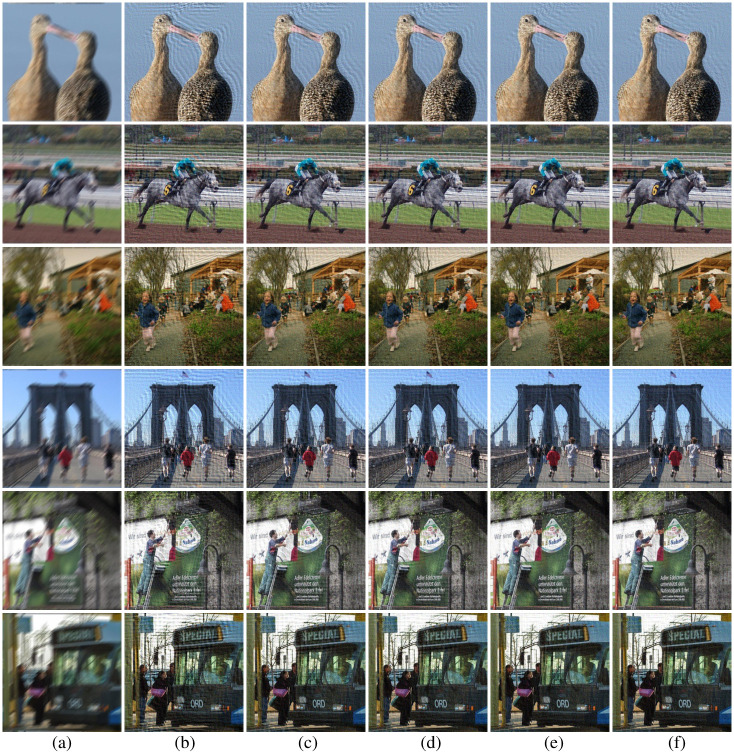
Deblurring results with different methods on VOC2012 dataset in noisy circumstances. (a) degraded image, (b) Moghaddam’s results, (c) Traditional-Radon-based results, (d) Dash’s results, (e) Wang’s results, (f) our results.

Apart from parameters estimation tests and experiments on noise-free images, we also conduct a set of experiments on noisy images and measure the PSNR and SSIM of the deblurred pictures to validate the robustness of our method. Similar to what we did in the Section 5.2, the images of VOC2012 dataset are blurred with different PSFs, and additive Gaussian noise with standard deviation *σ* = 0.0001 is added to produce noisy-blurred images (see also in Fig 12(a)). For fair comparison, blind deconvolution is used to deblur the degraded images by the detected PSFs with different methods. The visualization results for five methods are shown in Fig 12, and the PSNR and SSIM of the deblurred images with different approaches are expressed in Table 3 and Fig 11. From Fig 12, it can be observed that the deblurred images with Wang and our method exhibit better results than other three methods. Furthermore, through the PSNR and SSIM of the deblurred images produced by different methods in Table 3 and Fig 11, it can be seen that our method yields the largest PSNR and SSIM on VOC2012 database, since we ranked first in 96.76% of the VOC2012 dataset. These results demonstrate that the robustness of our method is superior to the other four methods in noisy circumstances.

**Table 3:** PSNR (dB) and SSIM of the deblurred images in noisy circumstances on VOC2012 database.

Method	Moghaddam [33]		Traditional Radon [25]		RBFNN [5]		Wang [12]		Our method	
Criterion	PSNR(dB)	SSIM	PSNR(dB)	SSIM	PSNR(dB)	SSIM	PSNR(dB)	SSIM	PSNR(dB)	SSIM
2007_000027.jpg	67.75	0.5889	68.96	0.6367	69.13	0.6428	69.84	0.6690	**70.13**	**0.6832**
2007_001733.jpg	69.61	0.5310	70.28	0.5627	70.39	0.5675	70.90	0.5891	**71.25**	**0.6047**
2007_005264.jpg	67.79	0.6435	69.01	0.6896	69.18	0.6960	69.88	0.7214	**70.15**	**0.7348**
2008_001454.jpg	68.45	0.3882	69.73	0.4339	69.93	0.4413	70.77	0.4744	**71.14**	**0.4931**
2008_001676.jpg	69.32	0.3807	70.10	0.4119	70.24	0.4177	70.83	0.4437	**71.20**	**0.4627**
2008_003974.jpg	68.77	0.7462	69.65	0.7723	69.77	0.7762	70.29	0.7916	**70.60**	**0.8016**
2008_004088.jpg	65.65	0.4244	67.30	0.5006	67.50	0.5099	68.22	0.5459	**68.40**	**0.5588**
2008_006397.jpg	67.59	0.4720	69.33	0.5439	69.59	0.5545	70.56	0.5956	**70.91**	**0.6135**
2008_007430.jpg	67.59	0.6328	68.83	0.6862	69.00	0.6928	69.67	0.7158	**69.93**	**0.7269**
2009_001117.jpg	66.89	0.5695	68.07	0.6407	68.24	0.6488	68.89	0.6767	**69.11**	**0.6860**
2009_003551.jpg	68.78	0.6646	69.79	0.7107	69.91	0.7164	70.48	0.7401	**70.75**	**0.7503**
2010_000109.jpg	68.79	0.7087	69.70	0.7408	69.83	0.7451	70.40	0.7620	**70.70**	**0.7712**
2011_001159.jpg	67.13	0.4843	68.48	0.5437	68.66	0.5518	69.37	0.5835	**69.60**	**0.5954**
2012_000279.jpg	66.13	0.5486	67.28	0.6085	67.41	0.6144	67.94	0.6388	**68.08**	**0.6465**
2012_002956.jpg	65.99	0.3825	67.34	0.4619	67.50	0.4709	68.15	0.5051	**68.32**	**0.5165**
2012_003170.jpg	68.01	0.5879	69.17	0.6511	69.34	0.6593	70.03	0.6913	**70.32**	**0.7044**
2012_004086.jpg	68.45	0.5184	69.57	0.5595	69.74	0.5660	70.48	0.5939	**70.81**	**0.6092**
*Overall*	67.83	0.5579	66.31	0.5867	69.25	0.6062	69.91	0.6305	**70.56**	**0.6571**

### 5.4 Summary: Experiments on our method

#### 5.4.1 High precision of blur parameters

Throughout the whole experiments, our method could achieve the best blur parameter estimation accuracy for both blur angle and blur length with the least mean absolute errors of 0.11° and 0.14 pixels.

#### 5.4.2 High quality of restoration

In the deblurring on noise-free images, we have the best results in 95.91% of the VOC2012 database on computational efficiency and deblurring quality, whereas in the noisy images we could achieve 96.76% best results in VOC2012 database.

All in all, one can see that the proposed method could estimate the blur parameters accurately and eliminate the influence from the linear motion effectively in the whole experiments.

## 6 Conclusions

An improved point spread function estimation approach for blind motion deblurring from the spectrum of a single image which is altered due to motion blur, noise and interference stripes was proposed in this research. To solve these problems in single blind image restoration, we propose a novel tri-Radon transforms blur angle estimation scheme which is inspired by the dark and bright stripes in frequency domain (spectrum). Based on this blur angle estimation approach the first order differential of image and auto-correlation matrix were combined to detect the blur length accurately. We performed a set of experiments on VOC2012 dataset and naturally motion blurred images to compare our proposed method against several state-of-the-art methods through the qualitative and quantitative assessments. The results of these experiments show that our method perform best with respect to different blur parameters and is proved to obtain satisfying deblurred images, since we could produce impressive accuracies for both blur angle and length with errors of only 0.11° and 0.14 pixels in the whole experiment respectively.

As there is almost no perfect linear motion between the camera and the scene in practical application, so it is worth to explore an effective solution for the nonlinear and nonuniform motion blur problem. In the future research we plan to improve our method work on nonlinear and nonuniform motion blurred images and extend our method to other datasets.
